# Formation of Sulforaphane and Iberin Products from Thai Cabbage Fermented by Myrosinase-Positive Bacteria

**DOI:** 10.3390/molecules23040955

**Published:** 2018-04-19

**Authors:** Vijitra Luang-In, Sirirat Deeseenthum, Piyachat Udomwong, Worachot Saengha, Matteo Gregori

**Affiliations:** 1 Natural Antioxidant Innovation Research Unit, Department of Biotechnology, Faculty of Technology, Mahasarakham University, Khamriang, Kantarawichai, Mahasarakham 44150, Thailand; sirirat.d@msu.ac.th (S.D.); u.piyachat@gmail.com (P.U.); worachot207@gmail.com (W.S.); 2 Department of Biology and Biochemistry, University of Bath, Claverton Down, Bath BA2 7AY, UK; mnjg20@bath.ac.uk

**Keywords:** antioxidant, cabbage, *Enterococcus*, *Enterobacter*, isothiocyanate, myrosinase

## Abstract

Myrosinase-positive bacteria from local fermented foods and beverages in Thailand with the capacity to metabolize glucosinolate and produce isothiocyanates (ITCs) were isolated and used as selected strains for Thai cabbage fermentation. *Enterobacter xiangfangensis* 4A-2A3.1 (EX) from fermented fish and *Enterococcus casseliflavus* SB2X2 (EC) from fermented cabbage were the two highest ITC producers among seventeen strains identified by 16S rRNA technique. EC and EX were used to ferment Thai cabbage (*Brassica oleracea* L. var. *capitata*) containing glucoiberin, glucoraphanin and 4-hydroxyglucobrassicin at 430.5, 615.1 and 108.5 µmol/100 g DW, respectively for 3 days at 25 °C. Different amounts of iberin nitrile, iberin, sulforaphane and indole 3-acetonitrile were produced by spontaneous, EX- and EC-induced cabbage fermentations, and significantly higher ITCs were detected (*p* < 0.01) with increased antioxidant activities. Iberin and sulforaphane production in EX-induced treatment peaked on day 2 at 117.4 and 294.1 µmol/100 g DW, respectively, significantly higher than iberin at 51.7 µmol/100 g DW but not significantly higher than sulforaphane at 242.6 µmol/100 g DW in EC-induced treatment at day 2. Maximum health-promoting benefits from this functional food can be obtained from consumption of a liquid portion of the fermented cabbage with higher ITC level along with a solid portion.

## 1. Introduction

Over the past 30 years, the public health landscape in Thailand has changed dramatically. Currently, non-communicable diseases (NCDs) are the leading causes of deaths in the country [1,2,3]. These include cancer, cardiovascular disease, emphysema, diabetes and cirrhosis which are thought to be associated with free radical induced oxidative damage [4]. Recently, attention has focused on the development of antioxidative supplements or foods containing antioxidants as an effective and natural way to diminish oxidative damage and exert a beneficial effect on human health [5,6]. 

Thai fermented cabbage (a.k.a. ka-lum-plee-dong) is a popular traditional Thai dish made from shredded white cabbage (*Brassica oleracea* L. var. *capitata*) fermented with naturally present lactic acid bacteria (LAB) in brined rice water for 1–3 days. It has similar characteristics to pao cai in China [7], sayur asin in Indonesia [8], kimchi in Korea [9] and sauerkraut in Europe [10]. White cabbage is enriched with glucosinolates (GSLs) 1 that differ between varieties, climatic and growth conditions [11]. When plant tissue is damaged during food preparation, the endogenous myrosinase enzyme (EC 3.2.1.147) comes into contact with GSLs and the subsequent hydrolysis produces isothiocyanates (ITCs), nitriles (NITs), thiocyanates and/or epithionitriles [12]. ITCs are the most prominent products, especially sulforaphane, which have proven chemopreventive, anti-inflammatory, antioxidant and immunomodulatory activities [13]. Microbial fermentation is commonly used to preserve vegetables, but it also enhances the antioxidant activity of sauerkraut possibly due to the synergistic effects of wounding and biochemical processes incurred by LAB [14]. 

Accumulating evidence suggests that certain bacterial strains with myrosinase activity including *Bifidobacterium* spp. [15], *Lactobacillus agilis* R16 [16], *Enterococcus casseliflavus* CP1 [16], *Escherichia coli* VL8 [16], *Citrobacter* sp. Wye 1 [17], *E. coli* O157:H1 [18] and *Enterobacter cloacae* [19] are capable of metabolizing GSLs to ITCs and/or NITs. To date, the myrosinase-positive bacteria have not been identified in Thailand.

Thus, this study aimed to isolate and identify myrosinase-positive bacteria from Thai fermented foods and use them to induce microbial fermentation of Thai cabbage. GSL degradation, ITC production and antioxidant activity in Thai fermented cabbages were investigated based on fermentation time and strains of bacteria used to induce fermentation. Results were compared with spontaneous fermentation and unfermented fresh cabbage. Traditionally, only the solid portion of Thai fermented cabbage is consumed while the liquid portion is discarded. Here, amounts of ITCs present in both portions were examined to ensure the maximum health benefits one can obtain from consuming Thai fermented cabbage. After consumption, bioactive ITC products exert positive effects inside the human body. Myrosinase-positive bacteria inhabit the gut and influence the bioavailability of GSLs and ITCs in ingested raw and cooked *Brassica* vegetables. Thus, Thai fermented cabbage can be promoted as a functional food with enhanced health-beneficial effects for the Thai population.

## 2. Results and Discussion

### 2.1. GSL-Metabolizing Bacteria from Thai Fermented Foods and Drinks

**Figure 1:**
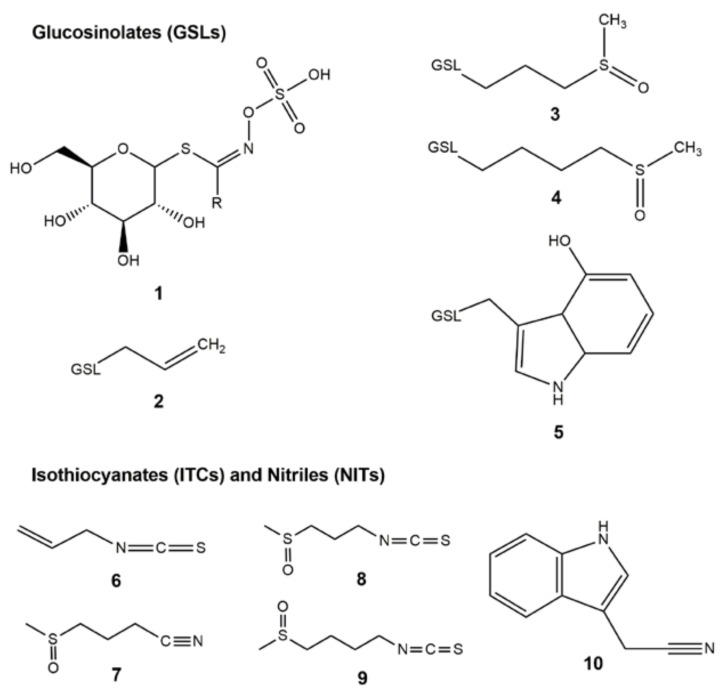
Chemical structures of GSLs **1**–**5** and ITC/NIT degradation products **6**–**10**. (**1**) Glucosinolate (GSL) core structure with R group; (**2**) sinigrin; (**3**) glucoiberin; (**4**) glucoraphanin; (**5**) 4-hydroxy-glucobrassicin; (**6**) allyl isothiocyanate; (**7**) iberin nitrile; (**8**) iberin; (**9**) sulforaphane; and (**10**) indole-3-acetonitrile.

Twenty-one bacterial isolates from eight sources of Thai local fermented foods and beverages were identified as GSL-metabolizing bacteria using selective M9 agar containing GSL called sinigrin **2** (Figure 1), and identified at subspecies level using 16S rRNA gene analysis. Results in Table 1 show the greatest number of newly identified GSL-metabolizing bacterial species coming from Thai fermented cabbage followed by Thai fermented fish, resulting in isolation of 8 and 3 bacterial species, respectively. When sinigrin was metabolized by bacterial myrosinase, the degradation product i.e., ITC namely allyl isothiocyanate (AITC) **6** was expected.

Most GSL-metabolizing bacteria were able to produce AITC from sinigrin metabolism using GC-MS and HPLC analyses, respectively. The majority of ITC-producing bacteria belonged to the genera *Enterobacter* and *Enterococcus*, with the two highest ITC producers named as *Ent. xiangfangensis* 4A-2A3.1 (EX) from fermented fish and *Ec. casseliflavus* SB2X2 (EC) from fermented cabbage producing 65 and 61 nmol AITC, respectively from 100% sinigrin degradation within 24 h. Therefore, these two isolates were chosen as starter cultures to ferment Thai cabbage in further experiments. Although the bacteria belonged to the same genus e.g., *Enterobacter*, they exhibited different GSL-metabolizing capacity, with possibly different myrosinase activity (Table 1). AITC was unstable in the culture medium [20], and AITC product formation never reached 100% with the highest found in EX at 65%. However, *Lactococcus hircilactis* WS16, *Lb. lactis* WS18 and *Bacillus* sp. KW3 did not produce AITC from 77–80% sinigrin degradation, suggesting that they may have different GSL metabolic enzymes, or utilize mechanisms from other bacteria to metabolize GSL but not to produce ITC.

Two bacterial species were found in more than one sample; *Enterobacter* sp. 1A-1A with 92% identity to *Enterobacter* sp. Md1-53 was found in fermented cabbage, pickled onions and fermented juices, while *Ent. faecalis* 5A-2B with 99% identity to *Ent. faecalis* NW A20 was present in both fermented cabbage and fermented herbal drink. Thus, 17 bacterial strains were identified from 20 isolates from eight Thai fermented food/drink samples. None of these 17 bacterial strains at subspecies level have ever been reported as GSL metabolizers and/or ITC producers, yet they shared the same genus or the same species as previously identified ones. Prior findings showed a variety of GSL-metabolizing bacterial strains such as *Bacillus thuringiensis* [21], Actinomycetes isolated from cotton soil [22], *E. coli* VL8, *Ec. casseliflavus* CP1 isolated from human faeces [23], *Lb. plantarum* KW30, *Lc. lactis* subsp. lactis KF147, *E. coli* Nissle 1917 isolated from foods [24], *B. pseudocatenulatum*, *B. adolescentis*, *B. longum* [15], *Lb. agilis* R16 [25], and a known myrosinase-producer *Ent. cloacae* isolated from soil [19]. 

Here, all the 17 identified bacteria belonged to the genus *Enterobacter* including *Bacillus*, *Lactococcus*, *Escherichia*, *Enterobacter* and *Enterococcus*. Contrary to popular belief, *Lc. hircilactis* WS16 (100% identity to *Lc. hircilactis* DSM 28960) and *Lc. lactis* WS18 (98% identity to *Lc. lactis* RCB787) did not produce ITC from GSL metabolism, indicating that not all LAB are able to produce ITC as previously thought. Similarly, it was found that *Lb. plantarum* KW30 and *Lc. lactis* subsp. lactis KF147 did not produce ITC from glucoraphanin, glucoerucin and glucoiberverin. Instead, they generated sulforaphane nitrile as well as erucin nitrile and iberverin nitrile [24]. 

Most isolated bacteria show highest % identity to the closest relative bacteria originating from food sources and plants, followed by human/animal guts and environments located mostly in Asia including China, India, Korea, Pakistan, Thailand and also other parts of the world including Italy, Belgium, Brazil and South Africa (Table 1). Probiotic reports of both *Enterobacter* spp. and *Enterococcus* spp. are very few; however, the genome of *Ent. xiangfangensis* isolated from Chinese traditional sourdough was recently published [26]. In addition, *Ec. faecium*-group and *Ec. faecalis*-group were also isolated at the early stages of cauliflower fermentation [27], indicating that *Enterococcus* and *Enterobacter* are commonly found in fermented foods.

### 2.2. Phylogenetic Tree of GSL-Metabolizing Bacteria

**Figure 2:**
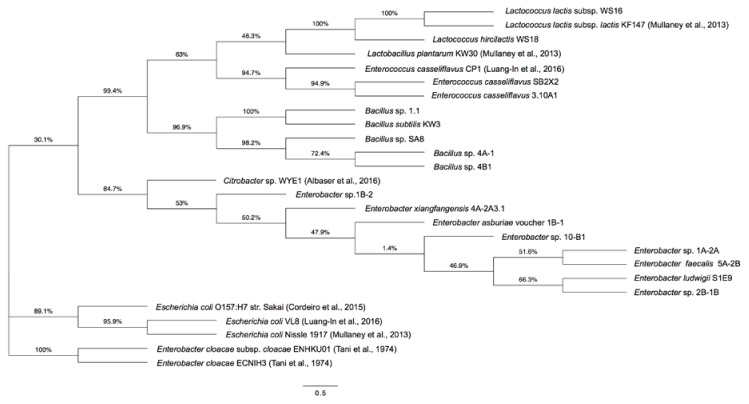
Phylogenetic tree of GSL-metabolizing bacteria isolated in this study and previous reports [16,17,18,19,24], inferred from 16S rRNA partial sequences from different bacteria using the Maximum Likelihood method based on the Le and Gascuel 2008 model. Percentage of replicate trees with associated taxa clustered together after bootstrapping (1000 replicates) is shown next to the branches. Horizontal bars represent a distance of 0.5 substitutions per site. Evolutionary analyses were conducted by MEGA7 and the phylogenetic tree was drawn using FigTree.

The phylogenetic tree shows 17 GSL-metabolizing bacteria isolated in this study and nine reference bacteria with GSL-metabolizing capacity from previous reports, categorized into three main groups (Figure 2). 

The first and biggest group (30.1% node) comprised all bacteria isolated here including LAB, *Enterococcus* and *Bacillus* along with the reference LAB [24] and *Enterococcus* [16] from previous findings. The subgroup consisted of *Enterobacter* as well as reference *Citrobacter* [17]. The second group (89.1% node) included only three reference *E. coli* bacteria [16,24,28]. Similarly, the third group (100% node) included only two reference *Enterobacter* bacteria [19]. Thus, *Enterobacter* spp. isolated here were evolutionarily more closely related to *Citrobacter* sp. WYE1 [17] than *Ent. cloacae* [19]. This is the first report identifying *Ent. xiangfangensis*, *Ent. ludwigii*, *Ent. asburiae* and several new *Bacillus* spp. as GSL metabolizers and also ITC producers.

### 2.3. ITC Products from Fermented Cabbage

**Figure 3:**
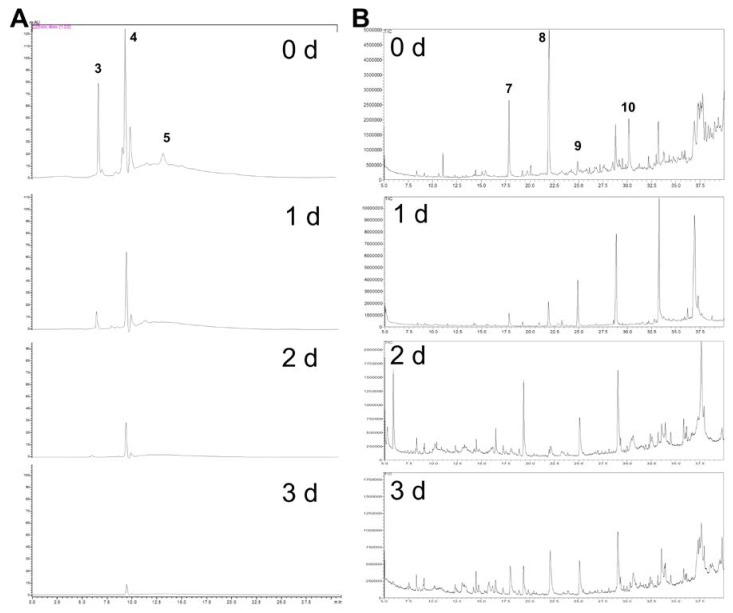
Substrates and degradation products in Thai cabbage fermentations over 3 days. (**A**) HPLC chromatograms showing GSL profiles of fermented cabbage by EX from 0, 1, 2, 3 days (0 d, 1 d, 2 d, 3 d); (**B**) GC-MS chromatograms showing metabolic profiles of fermented cabbage by EX from 0, 1, 2, 3 days (0 d, 1 d, 2 d, 3 d). (**3**) Glucoiberin at 6.30 min; (**4**) Glucoraphanin at 9.44 min; (**5**) 4-hydroxyglucobrassicin at 12.5 min; (**7**) Iberin nitrile at 17.9 min; (**8**) Iberin at 24.9 min; (**9**) Sulforaphane at 28.9 min; and (**10**) Indole-3-acetonitrile at 30.2 min.

The inoculums of EX and EC were used to ferment Thai cabbage containing three GSLs, namely glucoiberin (GIB), glucoraphanin (GRP) and 4-hydroxyglucobrassicin (GBS) at 430.5, 615.1 and 108.5 µmol/100 g dry weight, respectively (Table 2; Figure 3A) for 3 days at 25 °C. 

In comparison, Chinese cabbage (in kimchi) contained five types of GSLs at approximately 8.3 µmol/g dry weight including glucoalyssin, gluconapin, glucobrassicanapin, glucobrassicin, and 4-methoxyglucobrassicin [29]. The total GSLs including GIB, GRP and GBS in this work were present in higher amounts, indicating that cabbages in different countries contain various kinds of GSLs (Table S3) in diverse amounts depending on environmental factors such as geographical location, temperature, solar radiation, humidity and climatic conditions [30]. Four degradation products of the three GSLs namely iberin nitrile (IBN), iberin (IBR), sulforaphane (SFN) and indole 3-acetonitrile (IAN), respectively have already been detected in fresh cabbage but in smaller amounts to fermented samples (Figure 3B, Table 2). Product generation results from intrinsic plant myrosinase in cabbage contacting with GSL from tissue damage during the handling process when GSL hydrolysis occurs. Table 2 shows gradual GSL degradations in all treatments. Spontaneous cabbage fermentation produced less IBR and SFN at days 2–3 although high GSL degradation was observed suggesting that certain bacterial strains naturally present in the spontaneous fermentation may be able to degrade GSLs but are not capable of producing ITCs.

However, IBN was found in reduced amounts at days 2–3 for induced fermentation of cabbage than for spontaneous fermentation, possibly due to the presence of bacterial enzymes transforming nitrile products to other metabolites. GRP was degraded more rapidly during bacterial-induced cabbage fermentations than spontaneous fermentation, indicating the presence of specific GRP-metabolizing bacteria in the samples. GBS totally disappeared at the end of day 1 due to its initial low content in Thai cabbage (Figure 3A; Table 2)*.* Degradation products namely IBN, IBR, SFN and IAN were also detected in fresh cabbage (Figure 3B).

IAN production in all treatments was similar at each day and gradually declined over time. Production of both IBR and SFN in EX-induced cabbage fermentation peaked on day 2 at 117.4 and 294.1 µmol/100 g dry weight, respectively, significantly higher than IBR at 51.7 µmol/100 g dry weight but not significantly higher than SFN at 242.6 µmol/100 g dry weight in EC-induced fermented cabbage at day 2. Overall degradation products in all treatments declined over 3 days, never reaching 100% product formation (Table S2). This was perhaps caused by the unstable nature of ITCs in fermentation matrices [20] and a possibility that ITCs may kill the myrosinase-positive bacteria which then cannot produce the corresponding compounds. In addition, SFN content detected in the liquid portion of EC-induced cabbage fermentation at day 2 was significantly higher (*p* < 0.01) than found in the fermented cabbage solid portion by almost three-fold (Table 2). Typically, Thai people only consume a solid portion of fermented cabbage and discard a liquid portion with higher ITC level. Thus, this study should urge Thai consumers to consume both portions to obtain a maximum health-benefits provided by Thai fermented cabbage.

The pH values of fermented cabbages at day 3 were 3.55 (N), 3.25 (EX) and 3.45 (EC) with no statistical differences (Figure S2), comparable to pH values of 3.27–3.67 of sauerkraut from Spanish cabbage over 7-day fermentation at 25 °C by *Lb. plantarum* (CECT 748) and *Leuconostoc mesenteroides* (CECT 219) (Table S3). 

Similarly, ITC products including SFN at 39–49 µmol/100 g dry weight, IBR, and IBN were detected from metabolism of glucoraphanin and glucoiberin in sauerkraut from Spanish cabbage, similar to our products but in smaller amounts, except for allyl isothiocyanate (AITC) and allyl nitrile (ANIT) that were only found in sauerkraut. However, these products were not detected in their raw cabbage [31] suggesting that fermentation was responsible for ITC and NIT production. Similarly, sauerkraut made from cabbage grown in Finland produced SFN, AITC, ANIT and indole 3-carbinol (I3C) from glucoiberin, sinigrin and glucobrassicin, respectively [32].

In contrast, sauerkraut made from cabbage grown in Germany only produced ascorbigen and I3C from glucoiberin, sinigirin, glucobrassicin, glucoraphanin and 4-methoxy glucobrassicin [33]. SFN was also found in Korean kimchi but in reduced amount than fresh cabbage [34], and sometimes not found at all [35]. Different ITC production from diverse fermented cabbage products in various countries may result from the disparate cabbage cultivars used and bacterial species present in the fermentation.

### 2.4. Antioxidant Activity of Fermented Cabbage

**Figure 4:**
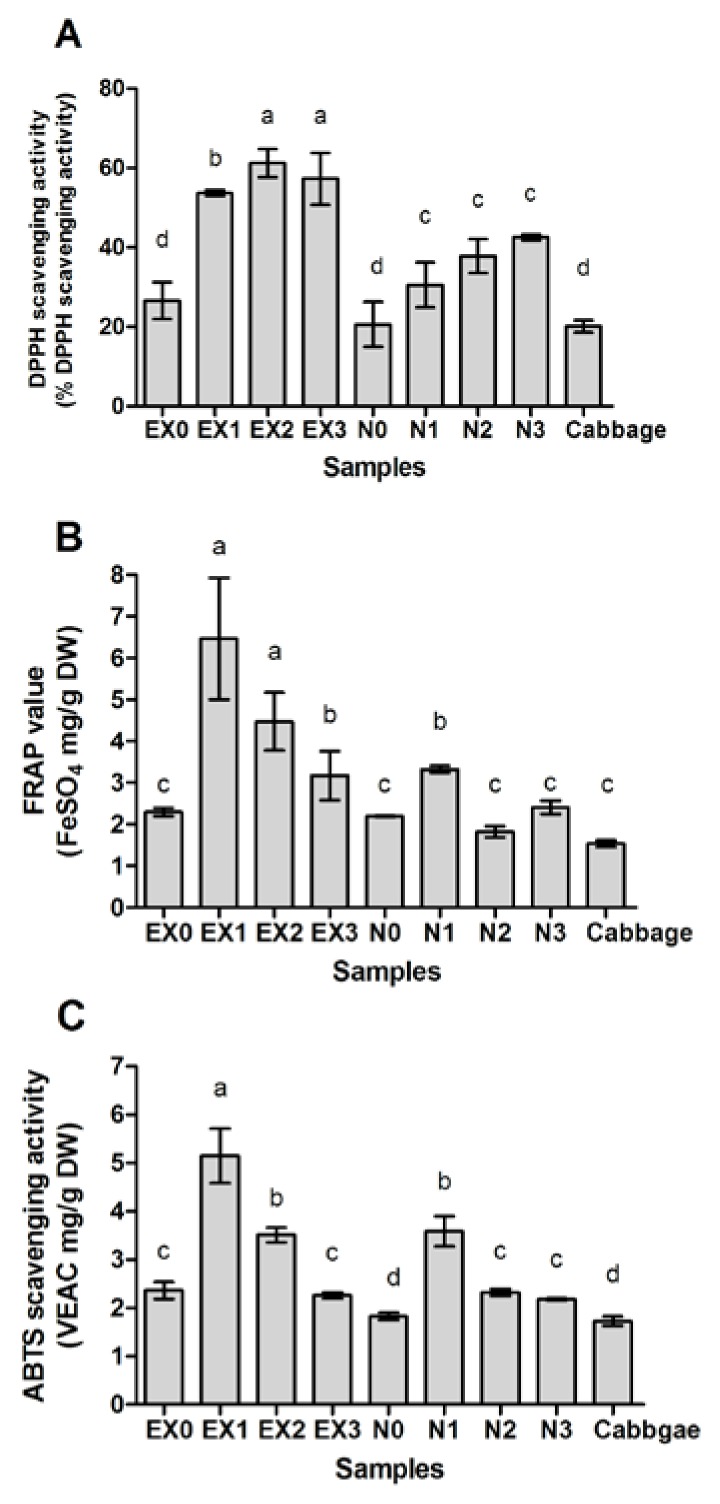
Antioxidant activities from fermented cabbage with/without bacterial induction over 3 days. (**A**) DPPH scavenging activity. Antioxidant activity was expressed as % DPPH scavenging activity; (**B**) FRAP value. Antioxidant activity was expressed as FeSO_4_ mg/g DW; (**C**) ABTS radical scavenging activity. Antioxidant activity was expressed as vitamin C equivalent antioxidant capacity (VEAC) mg/g DW. EX = Fermented cabbage induced with EX for 0, 1, 2, 3 days (EX0, EX1, EX2, EX3); N = Non-induced with EX (spontaneous fermentation of cabbage) for 0, 1, 2, 3 days (N0, N1, N2, N3); Cabbage = Fresh cabbage without fermentation. Different small letters (a–d) above the bars indicate significant differences (*p* < 0.01) according to Duncan’s multiple range test.

Since EX-induced cabbage fermentations yielded the highest ITCs, these were used to evaluate antioxidant activity compared with spontaneous fermentation. Results showed that over 3 days EX-induced cabbage fermentations exhibited significantly higher % DPPH scavenging activity (Figure 4A), FRAP activity (Figure 4B) and ABTS radical scavenging activity (Figure 4C) than spontaneous fermentations. 

Antioxidant activities of fresh cabbage at day 0 were similar to both spontaneous and induced fermentations and increased over time, indicating that bacterial metabolism was responsible for increasing antioxidants and ITCs in the fermented cabbage (Table 2).

Lactic-fermented cabbage in China, prepared by a dry-salt method and extracted with methanol, showed antioxidant activity of DPPH radical scavenging effect at 60% [36], similar to DPPH radical scavenging effect at 62.1% at day 2 by EX-induced fermentation (Figure 4A).

In addition, lactic-fermented red cabbage sprouts gave significantly higher antioxidant functionalities than their unfermented/control counterparts. Fermented red cabbage sprouts inoculated with *Lb. plantarum* showed the highest antioxidant activities (DPPH scavenging: 70.92%; TEAC: 1.94 mM Trolox equivalent), and almost two-fold higher than unfermented treatments.

Another possibility is that during fermentation, certain strains of *Lactobacillus*, *Enterococcus* and *Enterobacter* are able to produce exopolysaccharides (EPSs) with antioxidant potential from sugars and carbohydrates in cabbage [8] and thus enhancing antioxidant capacity in comparison with the unfermented cabbage. Cabbage is known to provide relatively higher fermentability than other vegetables because it has more fermentable saccharides [36]. For example, *Lb. acidophillus* and *Lb. bulgaricus* isolated from cabbage were found to be able to produce EPSs with 78.13–87.55% and 68.56–75.13% DPPH scavenging activities, respectively [37]. In addition, EPS from *Ec. faecium* K1 isolated from kalarei exhibited substantial DPPH scavenging ability (31.65–64.22%) at different concentrations of EPS (0.5–2.5 mg/mL) [38]. EPS from *Enterobacter* sp. YG4 isolated from the gut contents of the slug showed 25% hydroxyl radical scavenging ability and total antioxidant capacity in vitro at 32.5 µg VEAC/mL [39]. Antioxidant capacity of bacterial EPS might be attributed to its hydroxyl group and other functional groups in EPS, such as –COOH, C=O and –O–, which can donate electrons to reduce the radicals to a more stable form, or react with the free radicals to terminate the radical chain reaction [40]. These results indicated that fermentation using EPS-producing bacterial strains such as certain LAB and Enterobacter could be applied as a method to improve the potential antioxidant activities of fermented vegetables [41].

## 3. Materials and Methods

### 3.1. Sample Collection

Six out of eight fermented foods and beverages samples were purchased from local markets in Mahasarakham Province, Thailand for isolation of myrosinase-positive microbes. Samples (Figure S1) included (1) fermented cabbage; (2) picked onions; (3) fermented fish; (4) fermented pork; (5) fermented herbal drink; (6) fermented star fruit juice; (7) water kefir from Nakhon Ratchasima Province; and (8) milk kefir from Kamphaeng Phet Province. Samples were stored at 4 °C and analyzed within 24 h.

### 3.2. Isolation of GSL-Metabolizing Microbes

Solid food materials and liquid (5 g each, 10 g in total) or liquid beverage (10 mL) were weighed and mixed with 90 mL of sterile 0.85% NaCl solution. The mixture was homogenized in a sterile mortar and pestle for 5 min, mixed by vortexing for 5 min and then centrifuged at 4000 g for 15 min to obtain a clear supernatant. Enrichment culture technique was followed by inoculating 100 µL bacterial suspension into 900 µL LB broth containing 1 mM sinigrin for 2 days in an anaerobic incubator; this step was repeated at day 4, 6 and 8 in fresh Luria-Bertani (LB) medium (10 g Tryptone; 10 g NaCl; 5 g yeast extract in 1 L). At day 10, 100 µL of bacterial suspension was spread onto selective M9 minimal medium (1 M MgSO_4_; 1 M CaCl_2_; 50% glucose; 1% thiamine; 64 g Na_2_HPO_4_-7H_2_O; 15 g KH_2_PO_4_; 2.5 g NaCl; 5.0 g NH_4_Cl; 15 g agar in 1 L) containing 1 mM sinigrin and 2.5 mM barium acetate and incubated at 37 °C for 72 h in an anaerobic incubator. Growth and opaque zone formation were indicators of sinigrin degradation as evidenced by the white precipitation of barium sulfate. This demonstrated release of the sulfate group of GSL and, thus, GSL-metabolizing/myrosinase-positive isolates were selected from each food sample. Stock positive isolates were stored in LB medium with 20% glycerol at −80 °C. All microbial isolates were deposited in the Natural Antioxidant Innovation Research Unit, Department of Biotechnology, Faculty of Technology (WDCM 1160), Mahasarakham University, Thailand.

### 3.3. In Vitro Sinigrin Incubation

Sinigrin (1 mM) **2** was incubated with each selected bacterial culture (100 µL, OD_600nm_ = 0.5) from the previous step in 100 µL LB medium at 37 °C without shaking in an anaerobic incubator for 24 h. Bacterial cultures were centrifuged at 16,000 g for 5 min, then 100 µL of clear supernatant was removed for high performance liquid chromatography using a diode-array detector (HPLC-DAD), with the remaining 900 µL kept at −20 °C until required for gas chromatography-mass spectrometry (GC-MS) analysis.

### 3.4. Genomic DNA Isolation and 16S rRNA Gene Analysis

Selected isolates with the confirmed positive results of GSL degradation from HPLC analysis were cultured overnight for gram-staining, genomic DNA extraction, and PCR-based 16S rRNA gene analysis using universal primers following the previous method [23]. The phylogenetic tree of 16S rRNA partial sequences was constructed using FigTree software (v1.4.2) (Molecular evolution, phylogenetics and epidemiology, Edinburgh, Scotland, UK) [http://tree.bio.ed.ac.uk/software/ figtree/].

### 3.5. Starter Culture Preparation

Both selected bacteria were grown in 10 mL LB medium overnight and centrifuged at 8000 g for 15 min at 4 °C. Cells were washed twice with fermented sticky rice water (initial pH 6.0) overnight. A concentration of 10^6^ CFU/mL was inoculated in 3% (*v*/*v*) into the prepared cabbage-rice water jar (200 mL) and fermented as mentioned below. Induced fermentations (EX and EC) were defined by inoculation of either EX or EC culture and spontaneous fermentations (N = Non-induced) were those without inoculation of either culture.

### 3.6. Cabbage Fermentations

Thai white cabbage heads were purchased from Pran Fresh Co. Ltd., Khon Kaen, Thailand. After removing the core and outer layers, 3 kg of cabbage heads from the same batch were separated into leaf pieces manually according to the local cabbage fermentation procedure. Spontaneous fermentation was performed by thorough mixing of torn plant materials with 7% salt (*w*/*v*) and washing with distilled water. A total of 200 g solid plant materials were transferred into each replicate fermentation pot (500 mL glass container with lid, already containing 200 mL fermented rice water pH 6.0 mixed with 7% salt (*w*/*v*)). The salted cabbage materials were tightly pressed into the jars which were then closed and kept at 25 °C for 3 days without shaking. Triplicate measurements were performed throughout the study. The control was 200 g fresh cabbage heads separated into leaf pieces manually without fermentation at 0 h, and these were determined for initial GSL and ITC products and compared with spontaneously fermented cabbage samples (N) and cabbage fermentations induced by EX or EC.

### 3.7. Sampling and Extraction of Fermented Cabbage

Fermentation was carried out in parallel in 36 jars with triplicate measurements for each of the three treatments (N, EX and EC) from day 0 to day 3. Sampling was performed at day 0, 1, 2 and 3 with pH measured immediately after opening the fermentation jars. For extraction of GSLs and ITC products, the whole samples from each jar collected as mentioned above were frozen at −80 °C, dried in a freeze dryer and processed accordingly for GSL and ITC analyses as described below. For EC samples at day 2, half of the cabbage leafy material and half the fermented liquid were separated for GSL and ITC determination to evaluate which contained higher contents. Dried samples were ground into small pieces using a sterile mortar and pestle and weighed for extraction by 95% ethanol at concentration of 25 mg/mL at 25 °C for 24 h. The mixtures were centrifuged at 16,000 g for 5 min and clear supernatants were collected for antioxidant activity analyses.

### 3.8. Sample Preparation and HPLC Analysis to Detect GSLs

The GSL extraction method followed the previous report [42] with minor modifications. Freeze-dried samples (5 g) were ground and mixed with 5 mL of 70% methanol by shaking at 37 °C for 5 min, and the supernatant was collected after centrifugation at 8000 g for 15 min. The remaining solid sample was re-extracted and the supernatant was mixed with the first extraction. The mixture was dried at 70 °C in an oven and the dried residues were dissolved in 1 mL of deionized water using a vortex. The 1 mL sample was processed in DEAE-25A anion exchange resin as previously described [23]. A HPLC-DAD system (Shimadzu, Kyoto, Japan) fitted with a Synergi 4 µm Hydro-RP 80A, 150 × 2 mm, 4.6 micron (Phenomenex Inc., Torrance, CA, USA) protected with a security guard column AQ C18 (4 × 3 mm) comprising of Shimadzu LC-20AC pumps and a SPD-M20A diode array detector were used for GSL analysis using the following gradient: Water (Solvent A)–Acetonitrile (Solvent B) gradient 2% B (15 min), 2–25% B (2 min), 25–70% B (2 min), 70% B (2 min hold), 70–2% B (2 min), and 2% B (15 min) at a flow rate of 0.2 mL/min at 35 °C. Eluent was monitored at A229 nm. Quantification of desulfoglucosinolate (DS-GSL) was achieved using known response factors for each GSL relative to an external standard (sinigrin **2**). Pure sinigrin (Sigma-Aldrich Co., St. Louis, MO, USA), glucoiberin (**3**), glucoraphanin (**4**), and 4-hydroxyglucobrassicin (**5**) (Cfm Oskar Tropitzsch, Germany) were purchased as standards (Figure 1).

### 3.9. Sample Preparation and GC-MS Analysis to Detect Degradation Products

Freeze-dried samples (500 mg) were mixed with dichloromethane (DCM, 3 mL) in test tubes with tight lids for 24 h at 250 rpm at room temperature. The mixtures were centrifuged at 16,100 g for 5 min and the supernatants (1 mL) were added with 0.5 g magnesium sulfate, mixed and then centrifuged at 16,100 g for 20 min. Clear supernatants were transferred into vials and kept at −20 °C awaiting GC-MS analysis. A Shimadzu QP2010 system and an Agilent HP-5MS (5% phenylmethylsiloxane, 30 m × 0.25 mm i.d.; film thickness, 0.25 μm) capillary column were used for ITC analysis. GC-MS analytic conditions were performed as previously reported [23]. Ion source temperature was 230 °C and the electron multiplier voltage was 70.1 eV. Authentic standards of AITC **6** and SFN **9** (Figure 1) were purchased from Sigma-Aldrich Co. Identification was based on retention time and fragment ions (Table S1). Quantification of degradation products was calculated using an external standard curve of AITC or SFN.

### 3.10. Antioxidant Activity of Fermented Cabbage

This was evaluated through the free radical scavenging effect on 2,2′-diphenyl-1-picrylhydrazyl (DPPH) radical [43], ferric reducing antioxidant power (FRAP) assay [44] and 2,2′-azino-bis 3-ethylbenzthiazoline-6-sulphonic acid (ABTS) radical scavenging assay [45] using 25 mg/mL freeze-dried extract dissolved in 95% ethanol as the starting solution.

### 3.11. Statistical Analyses

Triplicates were used for each treatment and results expressed as mean ± standard deviation (SD). Significant differences between means were calculated by an analysis of variance (ANOVA) and Duncan’s multiple range test at *p* < 0.01 using SPSS package version 19.0 (IBM, Armonk, NY, USA).

## 4. Conclusions

This is the first report to highlight the application of newly isolated myrosinase-positive bacteria to produce Thai fermented cabbage with enhanced ITC levels, antioxidant activities and thus possibly enhanced health benefits. Induced cabbage fermentation by EX or EC produced higher SFN and IBR contents than spontaneous fermentation of cabbage and unfermented fresh cabbage. Fermented liquid and solid cabbage portions should be combined for consumption at day 1 or 2, when the highest ITCs with less nitrile products provide the maximum health-promoting benefits. Myrosinase-positive bacteria isolated from Thai fermented foods can be used as a starter culture for traditional fermented cabbage and promoted as a functional food in low cost with bioactivities from ITC products, similar to fermented cabbage products from other countries such as sauerkraut and kimchi.

## Figures and Tables

**Table 1:** Twenty bacterial isolates with GSL-metabolizing capacity isolated from local Thai fermented foods and drinks.

No.	Accession no. ^a^	Species	Closest Relative Species ^b^ (% Identity)/Accession no. ^c^/Origin of Isolate ^d^	Sinigrin Degradation (nmol)	AITC Product (nmol)	% Product Formation ^e^
1. Fermented cabbage pH 3.87						
1	LC342980.1	*Enterobacter* sp. 1A-1A	*Enterobacter* sp. Md1-53 (92%) MF581459.1 *Paeonia ostii* root, China	73 ± 8	30 ± 5	41 ± 4
2	LC342981.1	*Enterobacter faecalis* 5A-2B	*Enterococcus faecalis* NW A20 (99%) MG543833.1 Raw meat, South Africa	78 ± 7	39 ± 11	50 ± 14
3	LC342982.1	*Enterobacter asburiae* 1B-1	*Enterobacter asburiae* voucher ST56 (100%) KT287073.1 Rumen, China	75 ± 11	33 ± 8	44 ± 6
4	LC342983.1	*Enterobacter* sp. 1B-2	*Enterobacter* sp. NU33 (96%) MG459258.1 Plant growth-promoting bacteria in sugarcane, Brazil	79 ± 5	41 ± 7	52 ± 8
5	LC342984.1	*Enterobacter ludwigii* S1E9	*Enterobacter ludwigii* HTP04 (100%) KX024731.1 Shrimp gut, India	90 ± 8	50 ± 9	56 ± 7
6	LC342985.1	*Enterococcus casseliflavus* SB2X2	*Enterococcus casseliflavus* HMF4406 (98%) KT984002.1 Jeotgal (salted fermented food), Korea	100 ± 0	61 ± 4	61 ± 6
7	LC342986.1	*Bacillus* sp. SA8	*Bacillus* sp. SK123 (97%) KU060226.1 Honey bee apiary, Thailand	79 ± 8	39 ± 7	49 ± 6
8	LC342987.1	*Bacillus* sp. 1.1	*Bacillus* sp. BDU13 (96%) JX847614.1 Fermented fish, India	87 ± 10	42 ± 11	48 ± 10
2. Pickled onion pH 4.81						
9	LC342980.1	*Enterobacter* sp. 1A-1A	*Enterobacter* sp. Md1-53 (92%) MF581459.1 *Paeonia ostii* root, China	73 ± 5	40 ± 5	55 ± 4
10	LC342988.1	*Enterobacter* sp. 2B-1B	*Enterobacter* sp. SR19 (100%) KF896099.1 Seawater sediment, Belgium	71 ± 0	39 ± 3	55 ± 6
3. Fermented fish pH 4.60						
11	LC342989.1	*Enterobacter xiangfangensis* 4A-2A3.1	*Enterobacter xiangfangensis* W31 (100%) KP813789.1 Storm water bacteria in two urban lakes, China	100 ± 0	65 ± 3	65 ± 4
12	LC342990.1	*Bacillus* sp. 4A-1	*Bacillus* sp. S42 (100%) JX293317.1 Crystal tuff, China	71 ± 6	40 ± 4	56 ± 1
13	LC342991.1	*Bacillus* sp. 4B1	*Bacillus* sp. SO5.17 (97%) KC867296.1 Mine drainage, Brazil	73 ± 4	42 ± 9	58 ± 13
4. Fermented pork pH 4.73						
14	LC342992.1	*Enterococcus casseliflavus* 3.10A1	*Enterococcus casseliflavus* JFL12 (100%) KT343156.1 Fiber-degrading bacteria in rumen of Tibetan yak, China	74 ± 13	35 ± 8	47 ± 4
5. Fermented herbal drink pH 2.80						
15	LC342981.1	*Enterobacter faecalis* 5A-2B	*Enterococcus faecalis* NW A20 (99%) MG543833.1 Raw meat, South Africa	78 ± 7	35 ± 6	45 ± 5
6. Fermented juice pH 2.93						
16	LC342993.1	*Enterobacter* sp. 10-B1	*Enterobacter* sp. DBM3 (97%) KT957440.1 *Plutella xylostella* larval gut, China	77 ± 5	34 ± 11	42 ± 16
17	LC342980.1	*Enterobacter* sp. 1A-1A	*Enterobacter* sp. Md1-53 (92%) MF581459.1 *Paeonia ostii* root, China	73 ± 10	34 ± 8	47 ± 7
7. Water kefir from Nakhon Ratchasima pH 5.94						
18	LC336444.1	*Lactococcus hircilactis* WS16	*Lactococcus hircilactis* DSM 28960 (100%) KJ201026.1 Goat milk, Italy	77 ± 4	Nd	na
19	LC336446.1	*Lactococcus lactis* WS18	*Lactococcus lactis* RCB787 (98%) KT260999.1 Bat guano, India	78 ± 3	Nd	na
8. Milk kefir from Kamphaeng Phet pH 5.23						
20	LC342994.1	*Bacillus subtilis* KW3	*Bacillus subtilis* MA-48 (93%) KX426648.1 Rhizospheric soil in desert, Pakistan	80 ± 9	nd	na

^a^ GenBank accession no. of our strains deposited on NCBI website (http://www.ncbi.nlm.nih.gov/ pubmed); ^b^ Closest relative species and identity (%) from BLAST search on NCBI website; ^c^ GenBank accession no. of closest relatives on NCBI website; ^d^ Origins of closest relative species i.e., where each bacterium was isolated from; ^e^ % product formation = [AITC product (nmol)/sinigrin degradation (nmol)] × 100%. nd = not detected; na = not available. Each isolate from fermented samples was cultured in LB medium containing 1 mM sinigrin for 24 h. After that sinigrin degradation and AITC production were determined by HPLC and GC-MS, respectively.

**Table 2:** Metabolism of GSLs and formation of ITCs and NITs in fermented cabbage with/without bacterial induction over 3 days.

Samples	Remaining GSLs (µmol/100 g Dry Weight)			Products (µmol/100 g Dry Weight)			
	GIB	GRP	GBS	IBN	IBR	SFN	IAN
Cabbage	430.5 ± 34.1aB	615.1 ± 30.0aA	108.5 ± 19.1aC	75.1 ± 26.4aC	13.3 ± 10.0bD	39.5 ± 16.3dC	49.1 ± 29.8abC
N0	273.8 ± 15.8bB	534.4 ± 30.4abA	30.2 ± 4.4bC	61.82 ± 28.4abC	11.2 ± 6.0bD	56.2 ± 26.0dC	52.4 ± 19.1abC
N1	103.8 ± 19.5cB	419.0 ± 26.5cA	0.0 + 0.0cE	43.3 ± 23.2abC	32.1 ± 3.9bD	135.2 ± 42.0abB	22.9 ± 4.7abC
N2	20.9 ± 12.0dB	217.5 ± 14.8deA	0.0 + 0.0cC	35.2 ± 15.4abB	11.57 ± 5.8bD	127.9 ± 60.0abA	15.8 ± 2.0bB
N3	2.9 ± 1.1dC	146.3 ± 27.7deA	0.0 + 0.0cD	21.6 ± 11.0bcB	8.6 ± 3.9bB	112.2 ± 52.0abA	10.7 ± 3.1bB
EC0	305.7 ± 29.8bB	514.2 ± 42.6bA	27.4 ± 6.9bD	64.3 ± 23.3abC	15.4 ± 5.9bD	45.6 ± 22.0dC	60.5 ± 21.8aC
EC1	91.8 ± 17.3cC	359.9 ± 31.8cdA	0.0 + 0.0cE	25.6 ± 12.6abD	35.2 ± 20.0bD	177.8 ± 42.0abB	22.5 ± 14.6abD
EC2	21.0 ± 9.3dB	211.0 ± 27.7deA	0.0 + 0.0cD	6.4 ± 2.1cC	51.7 ± 35.9abB	242.6 ± 40.0aA	14.7 ± 11.9abB
EC3	3.1 ± 0.7dD	111 ± 20.2fB	0.0 + 0.0cE	2.9 ± 0.8cD	33.6 ± 22.0bC	222.4 ± 42.0aA	16.4 ± 7.2abC
EX0	305.2 ± 13.0bB	536.6 ± 29.1abA	31.6 ± 7.8aC	65.2 ± 20.2abC	12.5 ± 4.0bD	48.6 ± 30.0dC	55.3 ± 20.2abC
EX1	74.3 ± 14.6cC	275.9 ± 19.deA	0.0 + 0.0cF	26.6 ± 12.3abD	117.4 ± 41.9aB	294.1 ± 44.0aA	7.7 ± 3.7bE
EX2	11.1 ± 6.4dD	160.8 ± 29.0eB	0.0 + 0.0cE	6.5 ± 3.8cD	78.2 ± 38.0abC	252.6 ± 46.0aA	16.3 ± 7.4abD
EX3	2.4 ± 0.6dD	85.3 ± 24.7fB	0.0 + 0.0cE	13.8 ± 3.1bcC	70.5 ± 39.9abB	244.7 ± 70.0aA	20.9 ± 8.8abC
EC2 solid	5.2 ± 1.6dC	93.3 ± 23.6fA	0.0 + 0.0cD	3.6 ± 2.3cC	19.2 ± 10.0bB	69.7 ± 34.0cA	5.9 ± 1.2bB
EC2 liquid	16.3 ± 7.5dC	117.7 ± 34.4fB	0.0 + 0.0cF	3.5 ± 0.7cE	30.8 ± 22.0bC	173.3 ± 48.0abA	10.4 ± 1.3bD

Cabbage = fresh cabbage; N = Non-induced with bacteria (spontaneous fermentation of cabbage) for 0, 1, 2, 3 days (N0, N1, N2, N3); EC = Fermented cabbage induced with EC for 0, 1, 2, 3 days (EC0, EC1, EC2, EC3); EX = Fermented cabbage induced with EX for 0, 1, 2, 3 days (EX0, EX1, EX2, EX3); EC2 solid = Solid portion of fermented cabbage by EC at day 2; EC2 liquid = Liquid portion of fermented cabbage by EC at day 2. Different small letters within the same column and capital letters within the same row indicate significant differences (*p* < 0.01) according to Duncan’s multiple range test.

## Supplementary Materials

The following are available online. Table S1. Mass spectral (MS) data of metabolites detected in this work. Table S2. Percentage (%) production of degradation products upon glucosinolate metabolism during cabbage fermentation with/without induced bacterial culture over 3 days. Table S3. ITC products from different fermented cabbage products from different countries. Figure S1. Origins of Thai fermented foods and beverages and their physical appearances. Figure S2. The pH values of fermented cabbage with/without bacterial induction over 3 days.
