# Supplementary material for: Formation of Sulforaphane and Iberin Products from Thai Cabbage Fermented by Myrosinase-Positive Bacteria
Source: Molecules. 2018 Apr 19;23(4):955. doi: 10.3390/molecules23040955 (PMC6017806; doi:10.3390/molecules23040955)
Supplement: Supplementary file 1 [file molecules-23-00955-s001.pdf]

*Supplementary Materials*

# Formation of Sulforaphane and Iberin Products from Thai Cabbage Fermented by Myrosinase-Positive Bacteria

**Vijitra Luang-In [1,*], Sirirat Deeseenthum [1], Piyachat Udomwong [1], Worachot Saengha [1] and Matteo Gregori [2]**

[1] Natural Antioxidant Innovation Research Unit, Department of Biotechnology, Faculty of Technology, Mahasarakham University, Khamriang, Kantarawichai, Mahasarakham 44150, Thailand; sirirat.d@msu.ac.th (S.D.); u.piyachat@gmail.com (P.U.); worachot207@gmail.com (W.S.)

[2] Department of Biology and Biochemistry, University of Bath, Claverton Down, Bath BA2 7AY, UK; mnjg20@bath.ac.uk

* Correspondence: vijitra.l@msu.ac.th; Tel.: +66-43-754-085 (ext. 1833)

**Table S1.** Mass spectral (MS) data of metabolites detected in this work.

| Substrate | Degradation products[a] | MS spectra data m/z | $T_R$ (min)* |
|---|---|---|---|
| Sinigrin (SNG, 2) | Allyl-ITC (AITC) | 99 ($M^+$), 72, 65 | 6.9 |
| Glucoiberin (GIB, **3**) | Iberin nitrile (IBR NIT) | 131 ($M^+$), 115, 69, 61 | 17.9 |
| | Iberin (IBR) | 163 ($M^+$), 130, 116, 100, 72 | 24.9 |
| Glucoraphanin (GRP, **4**) | Sulforaphane (SFN) | 177 ($M^+$), 160, 115, 72 | 28.9 |
| 4-Hydroxy Glucobrassicin (GBS, **5**) | Indole-3-acetonitrile (IAN) | 155 ($M^+$), 130, 101, 77, 51 | 30.2 |
| NA | Palmitic acid | 256 ($M^+$), 213, 129, 73, 60 | 33.3 |
| NA | Linoleic acid | 280 ($M^+$), 193, 151, 69, 55 | 36.9 |

*Retention time at which degradation product was eluted as detected by GC-MS analysis. NA, not available; n.d., not determined; ND, not detected.

**Table S2.** Percentage (%) production of degradation products upon glucosinolate metabolism during cabbage fermentation with/without induced bacterial culture over 3 days.

| Samples | % Production | | |
|---|---|---|---|
| | IBN + IBR | SFN | IAN |
| N0 | 46.6 | 69.6 | 92.5 |
| N1 | 17.0 | 79.2 | 30.3 |
| N2 | 11.4 | 43.0 | 23.8 |
| N3 | 7.1 | 34.1 | 19.1 |
| EC0 | 63.9 | 45.2 | 74.6 |
| EC1 | 18.0 | 69.7 | 39.2 |
| EC2 | 14.2 | 82.7 | 32.0 |
| EC3 | 8.5 | 73.1 | 15.1 |
| EX0 | 62.0 | 61.9 | 71.9 |
| EX1 | 40.4 | 86.7 | 7.1 |
| EX2 | 20.2 | 71.3 | 15.0 |
| EX3 | 19.7 | 52.1 | 19.3 |
| EC2 solid | 5.4 | 14.2 | 11.0 |
| EC2 liquid | 8.3 | 54.6 | 18.8 |

N = Non-induced with bacteria for 0-3 days (N0, N1, N2, N3); EC = induced with EC for 0, 1, 2, 3 days (EC0, EC1, EC2, EC3); EX = induced with EX for 0, 1, 2, 3 days (EX0, EX1, EX2, EX3); EC2 solid = Solid portion of fermented cabbage of EC at day 2; EC2 liquid = Liquid portion of fermented cabbage of EC at day 2.

**Table S3.** ITC products from different fermented cabbage products from different countries.

| Sample | GSL profiles | ITC products | pH at the end of fermentation | Fermentation method |
|---|---|---|---|---|
| Thai picked cabbage (white cabbage from Khon Kaen, Thailand) | Glucoiberin<br>Glucoraphanin<br>4-hydroxy glucobrassicin | Iberin nitrile (IBN)<br>Iberin (IBR)<br>Sulforaphane (SFN)<br>Indole-3-acetonitrile (IAN) | 3.25-3.55<br>(3 days at 25 °C) | Spontaneous and induced fermentation by *Ec. casseliflavus* or *Ent. xiangfangensis* (This work) |
| Sauerkraut (white cabbage cultivars Bronco and Megaton from Spain) | Sinigrin<br>Glucoiberin<br>Glucoraphanin | SFN (39-49 µmol/100g DW)<br>IBR<br>IBN<br>allyl nitrile (ANIT)<br>allyl isothiocyanate (AITC) | 3.27 - 3.67<br>(7 days at 25 °C) | *Lactobacillus plantarum* (CECT 748) and *Leuconostoc mesenteroides* (CECT 219) by the Spanish Type Culture Collection (CECT, Valencia, Spain) [31] |
| Sauerkraut (*Brassica oleracea* L. var. capitata cv. Lennox from Finland) | Sinigrin<br>Glucoiberin<br>Glucobrassicin | AITC<br>ANIT<br>SFN<br>I3C | 3.9<br>(3 days at 20 °C) | Spontaneous vs *Leuconostoc mesenteroides* and *Pediococcus dextrinicus* (1:1) [32] |
| Sauerkraut (*Brassica oleracea* L. var. capitata cv. Storema RZ and cv. Lennox from Germany) | Glucoiberin Sinigirin<br>Glucobrassicin<br>Glucoraphanin<br>4-Methoxy glucobrassicin | Ascorbigen (13.0 µmol/100 g FW)<br>I3C (4.52 µmol/100g FW) | 4<br>(9 days at 20 °C) | Spontaneous [33] |
| Korean kimchi (white cabbage from Korea) | NA | 2.2 ppm SFN (lower than fresh cabbage)<br>3-butenyl ITC<br>4-methylthiobutyl | 4.53<br>(3 days at 20 °C) | Spontaneous [34] |
| Korean cabbage (*Brassica campestris* L. ssp. *peckinensis*, cultivar; 'Winter pride') | Gluconasturtiin<br>Glucobrassicanapin<br>4-methyl-pentyl GSL<br>Gluconapin | 2-phenylethyl ITC<br>4-pentenyl ITC<br>4-methylpentyl ITC<br>3-Butenyl ITC | No fermentation | Fresh cabbage [35] |

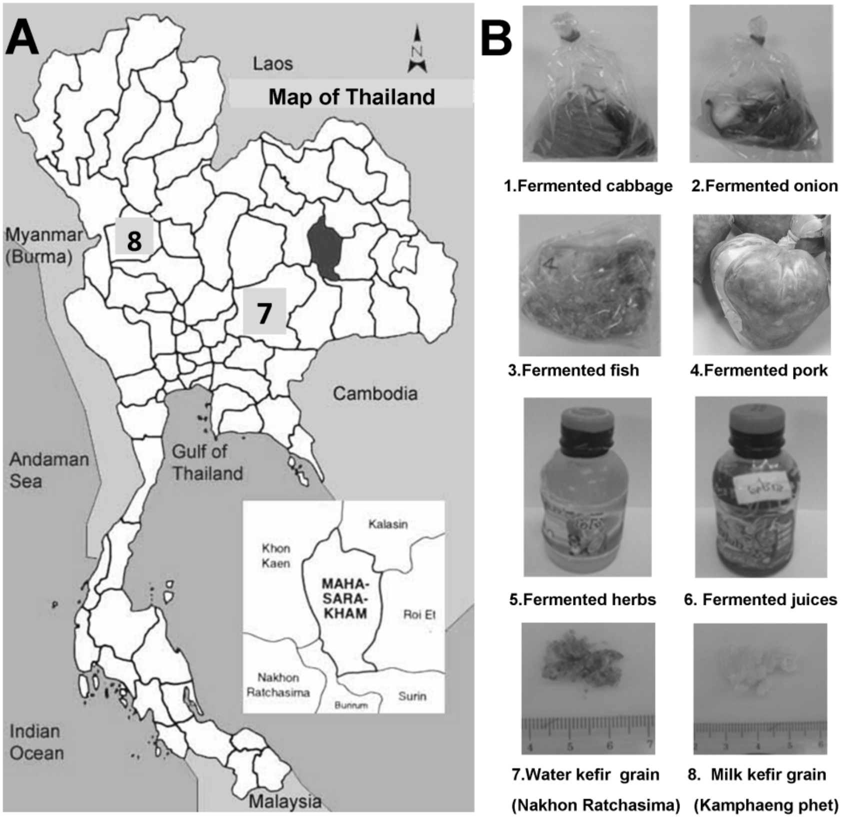


**Figure S1.** Origins of Thai fermented foods and beverages and their physical appearances. (A) Location of Mahasarakham Province where most fermented samples were collected and Nakhon Ratchasima and Kamphaeng phet. (B) Thai local fermented foods and beverages used in this study.

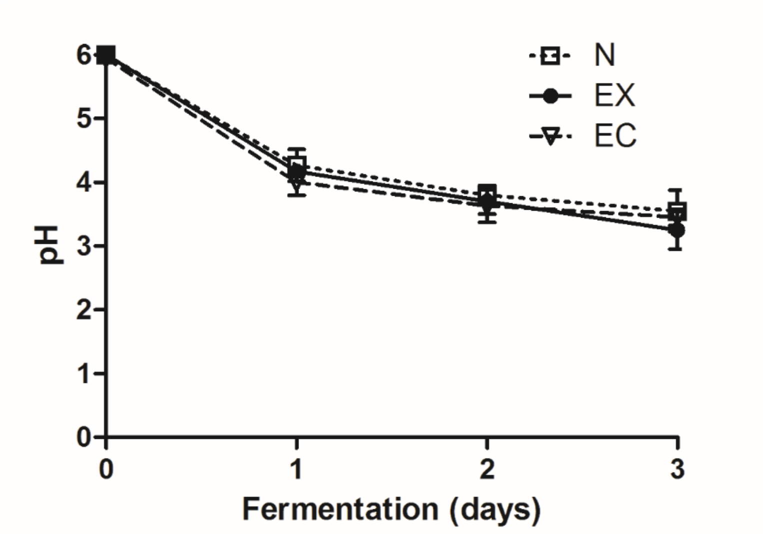


**Figure S2.** The pH values of fermented cabbage with/without bacterial induction over 3 days. N = Non-induced with bacteria; EC = induced with EC; EX = induced with EX.
